# Comparative study on the effects of Cr, V, and Mo carbides for hydrogen-embrittlement resistance of tempered martensitic steel

**DOI:** 10.1038/s41598-019-41436-2

**Published:** 2019-03-26

**Authors:** Junmo Lee, Taekyung Lee, Dong-Jun Mun, Chul Min Bae, Chong Soo Lee

**Affiliations:** 1 Pohang Research Lab, POSCO, Pohang, 37877 Korea; 2 School of Mechanical Engineering, Pusan National University, Busan, 46241 Korea; 3 Graduate Institute of Ferrous Technology, Pohang University of Science and Technology (POSTECH), Pohang, 37673 Korea

## Abstract

In this study, the ideal alloying element (among Cr, V, and Mo carbides) to enhance the resistance to hydrogen embrittlement (HE) in a tempered martensitic steel was investigated. Four types of steels were designed to contain cementites, Cr-rich M_7_C_3_ carbides, V carbides, and Mo carbides, respectively. These steels were tailored to possess a comparable tensile strength (~1.6 GPa). The HE resistances of these steels were evaluated through the slow strain rate test and cyclic corrosion test. The results showed an enhanced HE resistance, characterized by a high notch fracture strength after hydrogen charging, in the samples containing V carbides and Mo carbides. In particular, Mo carbide was regarded as the most ideal alloying element for HE resistance because of the high resistivity parameter, inhibited hydrogen penetration, and suppressed strength loss by internal hydrogen.

## Introduction

High-strength steels have been actively developed in the steel industry to secure competitiveness in the market. Such trends have raised the importance of the hydrogen embrittlement (HE) issue since high-strength steels, such as a tempered martensitic steel, are particularly susceptible to this phenomenon^1^. A high density of dislocations in the lath structure and film-like cementite in a tempered martensitic steel leads to high susceptibility to the HE^2,3^. Although tempering at elevated temperatures would reduce the dislocation density, there is a risk of deterioration in material strength. Such a risk can be alleviated by utilizing carbide precipitates formed above 500 °C that maintain the high strength of the steel even after applying a tempering process. There have been various reports of improvement of HE resistance and preservation of high strength in tempered martensitic steels using this approach^4–6^.

Despite the academic and industrial importance, however, there has been only limited research on the effect of carbide compositions and amounts on the HE properties of tempered martensitic steels. Wei *et al*.^7–11^ have conducted a series of valuable studies on this topic, particularly related to the effect of Nb, Ti, and V carbides on HE characteristics. According to their reports, these three types of carbides are nucleated at coherent interfaces in a nano-scale size. Their growth gradually changes the interfacial characteristic from being coherent, to semi-coherent, and to incoherent, which exerts a strong influence on the hydrogen-trapping behavior of each carbide. Takahashi *et al*.^12,13^ directly observed the occurrence of hydrogen trapping at the coherent interface of Ti and V carbides for the first time in 2010. Asahi *et al*.^4^ have investigated the hydrogen trapping and detrapping behaviors of V-added steels. Meanwhile, compared to V carbides, even less attention has been paid to Cr and Mo carbides. We have recently discussed the influence of these carbides on the HE resistance of a tempered martensitic steel in terms of their three variables, such as chemical affinity, solubility, and size^14,15^.

More importantly, the issue that still needs to be addressed is to determine *which* carbide is the best for enhancing HE resistance and strength simultaneously. It is difficult to find the answer based only on the previous studies because of the wide range of experimental conditions. There has been no research to directly compare Cr, V, and Mo carbides under the same condition thus far. Such a comparison is crucial for the industry as well as the academia because of the vast potential of these carbides that are liable to dissolve during the austenitizing step. Therefore, this study was aimed at investigating and comparing the effects of Cr, V, and Mo carbides on the HE resistance of a tempered martensitic steel. For this purpose, factors relevant to HE behavior other than carbides, e.g., film-like cementite and strength difference, were eliminated as much as possible. Furthermore, the microstructural evolution, hydrogen-trapping capability, and decrease in strength under two different HE conditions (i.e., cathodic precharging and cyclic corrosion test (CCT)) for four types of steels, were also investigated.

## Results

**Figure 1:**
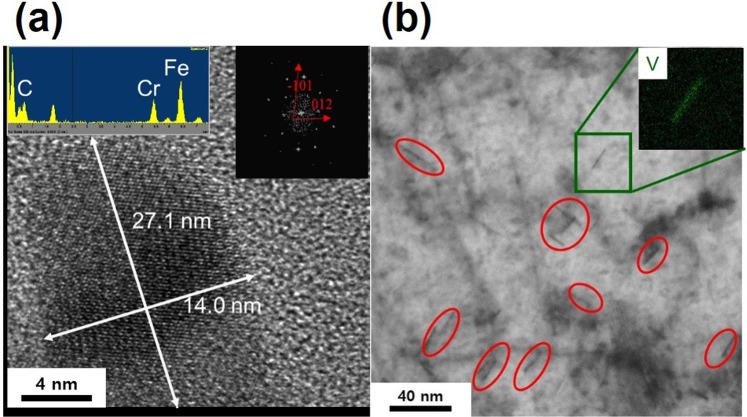
TEM micrograph of (**a**) M_7_C_3_ carbides in the replica sample of 2Cr steel and (**b**) FIB sample of 1Cr-0.2 V steel. The insets in (**a**,**b**) show the diffraction pattern and TEM/EDS mapping of V element, respectively.

Microstructural analysis using transmission electron microscopy (TEM) (Fig. 1) demonstrates the consistent results with the thermodynamic calculations (Fig. S1). Cementites (M_3_C) were the only type of precipitates confirmed in 1Cr steel^14^. This is consistent with the thermodynamic calculations that implied the absence of Cr-rich carbides (M_7_C_3_) when 1% Cr was added to the steel. In case of 2Cr steel, the increasing amount of Cr element gave rise to the formation of M_7_C_3_ carbides at the tempering temperature of 550 °C (Fig. 1a), as expected from the thermodynamic calculations. V carbides were thermodynamically expected to be stable at 570 °C in 1Cr-0.2 V steel on the basis of results shown in Fig. S1. Indeed, TEM/EDS mapping showed the formation of needle-like V carbides (Fig. 1b). These carbides were formed at right angles to each other owing to the Baker-Nutting orientation with a ferritic matrix (i.e. (001)VC//(001)*α* and [110]VC//[100]*α*)^16^. It is also worth noting a significantly smaller size of V carbides in 1Cr-0.2 V steel as compared to Cr carbides in 2Cr steel.

**Figure 2:**
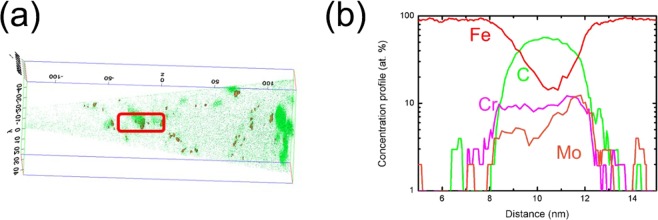
3DAP analysis of 1Cr-0.4Mo steel: (**a**) iso-surface image of 5-at.% Mo and (**b**) atomic concentration profile of Fe, C, Cr, and Mo in the marked area of (**a**).

The case of 1Cr-0.4Mo steel was more complex than that of other steels. The thermodynamic calculations indicated the presence of two types of carbides at 570 °C (i.e., a Cr-rich phase (M_23_C_6_) and Mo carbides) as shown in Fig. S1. The former did not precipitate under the current tempering condition since precipitation required a lot of time to grow^17^. This was further supported by TEM analysis that confirmed the absence of M_23_C_6_ particles in the present 1Cr-0.4Mo steel. The Mo carbides were too small to be observed by a conventional TEM measurement. Therefore, the three-dimensional atom probe (3DAP) method was adopted alternatively and demonstrated that Mo existed as either cluster or nano-sized (~4 nm) carbides (Fig. 2).

**Figure 3:**
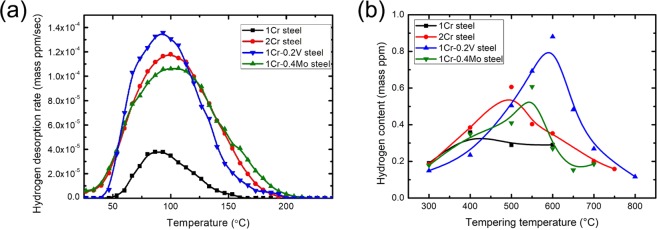
TDS analysis: (**a**) the comparison of TDS curves after hydrogen charging at a current density of 10 A∙m^−2^ for 48 h in 0.1% NaOH solution. The tempering conditions were presented in Table S1; (**b**) the variation in hydrogen content depending on the tempering temperature.

The formation of carbides had a strong influence on the hydrogen-trapping capability characterized by thermal desorption spectrum (TDS) curves (Fig. 3a). The capability decreased in order of 1Cr-0.2 V steel > 1Cr-0.4Mo steel > 2Cr steel ≫ 1Cr steel. The peak of the TDS curves was generated at a temperature of ~90 °C for all specimens. For further understanding, internal hydrogen content was presented in respect to an extensive range of tempering temperatures (Fig. 3b). The entire TDS curves were given in the supplementary information (Fig. S2). Wei *et al*.^8^ successfully interpreted the effect of Ti carbides on hydrogen-trapping behavior using this approach, which has been expanded to other carbides in this work. The hydrogen-trapping capability of 1Cr steel (i.e., alloy-carbide-free steel) rarely changed in the tempering range of 400–600 °C. In contrast, the other three steels exhibited a dramatic change in TDS curves depending on the tempering temperature. It is interesting to note that the marked change took place above 500 °C for these steels. Specifically, the maximum hydrogen-trapping capability was confirmed at tempering temperatures of 500, 600, and 550 °C for 2Cr steel, 1Cr-0.2 V steel, and 1Cr-0.4Mo steel, respectively.

The activation energy (*E*_*a*_) of each specimen was determined using the Kissinger’s equation^18^:

1 $$\partial \,\mathrm{ln}({\Phi }/{T}_{c}^{2})/\partial (1/{T}_{c})=-\,{E}_{a}/R$$

where *Φ* is the heating rate, *T*_*c*_ is the peak temperature, and *R* is the gas constant (Fig. S3). For data reliability, all samples were left exposed in the ambient atmosphere for 24 h in order to calculate the activation energy for only those hydrogens trapped at the carbides, and not for those in the matrix and grain boundaries. Asahi *et al*.^4^ have previously obtained the activation energy for V carbides using this method, and the present results are consistent with the reported data. The calculated values of activation energy were 14.1, 17.4, 27.6, and 21.5 kJ∙mol^−1^ for 1Cr steel, 2Cr steel, 1Cr-0.2 V steel, and 1Cr-0.4Mo steel, respectively.

**Figure 4:**
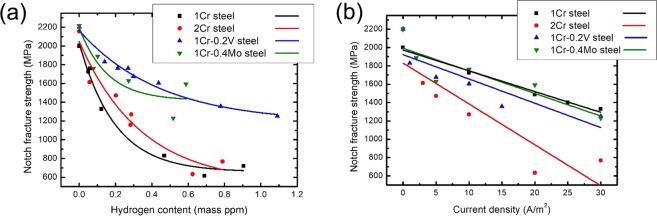
NFS variation in respect to (**a**) hydrogen content and (**b**) current density for the investigated steels.

**Figure 5:**
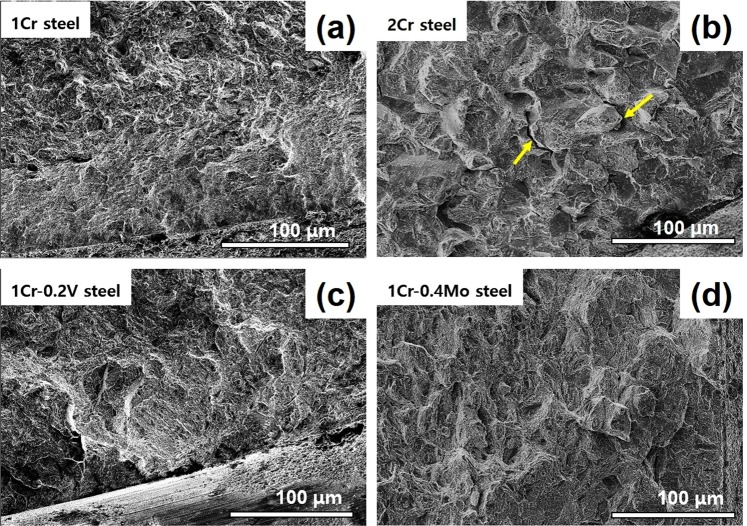
Fracture surface of the hydrogen-charged samples taken in the vicinity of notch root. The yellow arrows in (**b**) indicate the intergranular cracking.

The reduction of notch fracture strength (NFS) as a result of the HE phenomenon could be interpreted in two ways: hydrogen content inside a material (Fig. 4a) and current density applied in the charging step (Fig. 4b). In the former case, 1Cr-0.4Mo steel and 1Cr-0.2 V steel exhibited an improved HE resistance as compared to 1Cr steel and 2Cr steel. These results implied a beneficial effect of addition of V and Mo carbides on HE resistance. Note that this rank excludes the difference in the hydrogen-trapping capability among the investigated steels, since they were compared under the same hydrogen content. Meanwhile, 2Cr steel exhibited a marked deterioration in HE resistance when the data was rearranged with respect to the current density (Fig. 4b). These results are consistent with the fracture surface of 2Cr steel. The hydrogen-charged steels showed a dimple-fractured surface with a low area fraction of quasi-cleavage fracture, except for 2Cr steel that showed the intergranular fracture (Fig. 5). The trends are in good accord with hydrogen-charged steels in the literature^14,19,20^. In other words, the local area in 2Cr steel was particularly susceptible to the HE phenomenon, which rationalizes its poor HE resistance, as confirmed in both diagrams in Fig. 4.

In the present work, the concept of resistivity parameter (often called hydrogen susceptibility parameter) was adopted in order to quantitatively compare the HE resistances of the investigated steels. The parameter is defined as follows^21^:

2 $$([{H}_{C}]-[{H}_{E}])/[{H}_{C}].$$

where [*H*_*C*_] indicates the critical hydrogen concentration determined as the amount of hydrogen in a sample exhibiting 90% of ultimate tensile strength (UTS) (~1.44 GPa) compared to the hydrogen-uncharged material. [*H*_*E*_] is the maximum hydrogen content in the corrosion environment.

**Figure 6:**
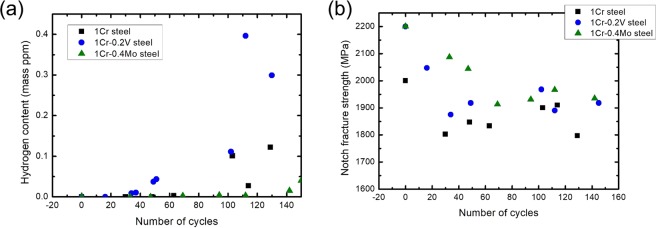
Variation in (**a**) hydrogen content and (**b**) NFS in respect to the number of cycles in CCT. The red circle in (**a**) indicates the highest amount of charged hydrogen among the investigated steels.

Internal hydrogen content and NFS were provided with respect to the number of CCT cycles for the investigated steels (Fig. 6). 2Cr steel was excluded from this analysis since results depicted in Fig. 4 had already confirmed that it showed the worst HE resistance. Interestingly, 1Cr-0.2 V steel exhibited a significantly high value of [*H*_*E*_] (0.4 ppm, as marked by the red circle in Fig. 6a) in contrast to the others. Considering Fig. 4a, a hydrogen content of 0.4 ppm should have reduced the NFS of this steel to ~1.6 GPa. However, the NFS decreased only to ~1.9 GPa after the CCT (as confirmed in Fig. 6b), suggesting a discordance between the slow strain rate test (SSRT) and CCT. This discordance will be explained later in terms of hydrogen-trapping sites in the section of *Discussion 3*.

## Discussion

### Effect of carbides on hydrogen-trapping capability

The increasing tempering temperature gave rise to a reduction in the dislocation density as well as the spheroidization of film-like cementites. Recalling Fig. 3b, the TDS curves of 1Cr steel rarely changed in the temperature range of 400–600 °C, indicating the negligible influence of dislocation density and cementite morphology on the hydrogen-trapping capability of this material. The microstructural evolution of 1Cr steel within this temperature range would not be enough to make a meaningful difference.

Meanwhile, the smallest TDS curve of 1Cr steel in Fig. 3a suggests that the precipitated carbides other than cementites enhanced the hydrogen-trapping capability. The marked change of TDS curves above 500 °C for the carbide-containing steels, as shown in Fig. 3b, could be understood by the fact that most carbides start precipitating at this temperature^22^. In other words, the hydrogen-trapping capability was radically improved above this temperature, and this conclusion was also supported by the previous literature reports^11,13^.

The hydrogen contents of 2Cr steel and 1Cr-0.2 V steel rapidly decreased with increasing temperature above 500 °C and 600 °C, respectively, as shown in Fig. 3b. This phenomenon arose from the growth of carbides, which made their interface incoherent^23^. This is consistent with our previous study reporting the loss of hydrogen-trapping capability owing to incoherent and undissolved V carbides^15^. 1Cr-0.4Mo steel showed a similar decrease in the TDS curves from 550 °C. This was attributed to a decreased fraction of Mo carbides, as confirmed in Fig. S1.

### Cr carbides

Recalling Fig. 4, 2Cr steel exhibited the worst HE resistance among the investigated materials from both viewpoints: hydrogen content and current density. This is contrasted by 1Cr steel that exhibited a deterioration in HE resistance only in terms of hydrogen content. The results indicate an adverse impact of Cr-rich M_7_C_3_ carbides on the HE resistance of a tempered martensitic steel.

2Cr steel showed the lower activation energy for hydrogen detrapping than that of the other steels containing V and Mo carbides. The results suggested that M_7_C_3_ carbides acted as a diffusible hydrogen-trapping site. Similar conclusions were proposed by Otsuka *et al*.^24^ utilizing tritium micro-autoradiography. It is also noted in Fig. 3a that 2Cr steel contained a markedly higher amount of hydrogen than 1Cr steel. A high concentration of diffusible hydrogen reduced the bonding force at grain boundaries and phase interfaces^25,26^, resulting in the decrease in NFS. In addition, the higher diffusivity of Cr in ferrite than the other carbide-forming elements caused a rapid coarsening of Cr-rich carbides. This is consistent with the lowest peak temperature of 2Cr steel, as shown in Fig. 3b, which indicates the rapid loss of hydrogen-trapping capability in Cr-rich carbides.

**Figure 7:**
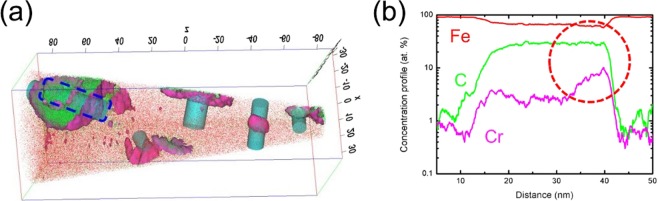
3DAP analysis of 2Cr steel: (**a**) iso-surface image of 4 atomic percent Cr and (**b**) atomic concentration profile of Fe, C, and Cr in the marked area of (**a**).

The interfacial state of the carbides, which affected the binding energy of hydrogen, also contributed to the negative effect of M_7_C_3_ carbides on HE resistance. As mentioned earlier, M_4_C_3_ carbides were precipitated in the form of plates while maintaining the Baker-Nutting orientation with respect to a ferritic matrix; most hydrogen atoms were trapped at misfit dislocations at this interface^13^. On the other hand, M_7_C_3_ carbides were precipitated at the interface between M_3_C particles and the ferritic matrix, and rapidly grew toward the M_3_C particles^27^. This type of precipitation was observed in 2Cr steel by 3DAP analysis (Fig. 7). The analysis confirmed a higher Cr concentration at the carbide surface rather than the carbide interior, as marked with a red circle in Fig. 7b. This result supports the aforementioned conclusion that the precipitation of M_7_C_3_ occurs at the M_3_C-ferrite interface. The absence of a special orientation between M_7_C_3_ carbides and ferritic matrix indicates an incoherent carbide interface. Most carbides trap hydrogen stably at coherent and semi-coherent interfaces^9^. Therefore, hydrogen in 2Cr steel was unstably trapped and liable to diffuse inside the material. This further assisted in the local concentration of internal hydrogen and resultant weakening of bonding force, leading to the worst HE resistance in 2Cr steel.

Recalling Fig. 5, 2Cr steel exhibited a unique fracture behavior among the investigated steels. Yoshino^28^ suggested a reduced resistance to stress corrosion cracking due to incoherent carbides. Song *et al*.^20^ attributed intergranular cracking to localized hydrogen concentration at grain boundaries. Yamaguchi *et al*.^26^ proposed the role of carbides in reducing interfacial strength between lath-grain boundaries. A similar mechanism was considered for the intergranular fracture in 2Cr steel as its Cr carbides had a large size and low activation energy for hydrogen detrapping.

### V carbides

There are two important issues regarding the HE phenomenon for the industry in terms of the resistivity parameter. The first issue is to prevent hydrogen from penetrating into a material under the applied conditions. This is interpreted in terms of the [*H*_*E*_] parameter. The second issue is to avoid a local concentration of hydrogen by trapping it in a stable manner, which is related to the [*H*_*C*_] parameter. The effect of V and Mo carbides is discussed in the following paragraph in view of these issues.

**Figure 8:**
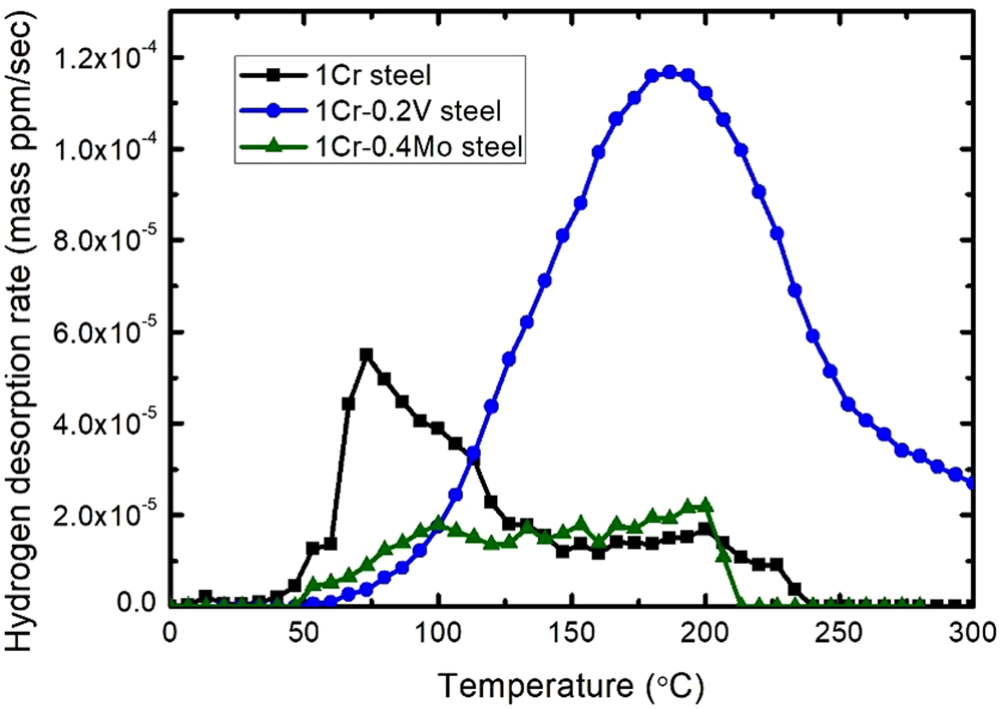
TDS analysis of 1Cr steel, 1Cr-0.2 V steel, and 1Cr-0.4Mo steel after applying 110 cycles of CCT.

Recalling Fig. 6b, the NFS of 1Cr-0.2 V steel decreased only to ~1.9 GPa during the CCT despite its high [*H*_*E*_] value. Such results can be understood by considering the hydrogen-trapping sites in this steel. 1Cr-0.2 V steel showed a peculiar TDS curve after 110 cycles of CCT (Fig. 8); this condition resulted in the highest amount of hydrogen being trapped inside a material (Fig. 6a). The peak temperature of 1Cr-0.2 V steel was obviously higher than the other two steels. Comparing Figs 3a and 8, it was evident that the peak temperature after the CCT increased by ~90 °C, whereas that of 1Cr steel rarely changed. 1Cr-0.4Mo steel showed an intermediate level of change in the peak temperature between these two steels. The obtained results imply a change in the major hydrogen-trapping sites depending on the HE condition (i.e., cathodic precharging or CCT) in 1Cr-0.2 V steel.

During the corrosion process, hydrogen penetrates into a specimen by the following chemical reactions:

3 $${{\rm{H}}}_{2}{\rm{O}}+{{\rm{e}}}^{-}={\rm{H}}+{{\rm{OH}}}^{-}\,({\rm{in}}\,{\rm{neutral}}\,{\rm{or}}\,{\rm{alkaline}}\,{\rm{solution}})$$

4 $${{\rm{H}}}^{+}+{{\rm{e}}}^{-}={\rm{H}}\,({\rm{in}}\,{\rm{acid}}\,{\rm{solution}})$$

The reaction shown in Eq. (3) occurs slowly because of a retarded supply of electrons as compared to that of hydrogen in a general corrosive situation. Accordingly, a hydrogen release from the specimen occurs much faster than hydrogen penetration in typical cases, including Cr steel and Cr-Mo steel.

A similar phenomenon would be expected to occur at dislocations and grain boundaries in 1Cr-0.2 V steel. However, in 1Cr-0.2 V steel, a significant amount of hydrogen was still trapped at the V carbides because of their stable hydrogen-trapping ability. In other words, V carbides acted as a non-diffusible hydrogen-trapping site^4^, which were distinguished from other trapping sites, such as dislocations. Both types of hydrogen-trapping sites contributed to the HE behavior after charging hydrogen cathodically, as shown in Fig. 3a. In contrast, it is only V carbides that mainly affected the HE behavior at the middle and late stages of CCT, as shown in Fig. 6, since hydrogen escaped from the diffusible hydrogen-trapping sites over time. Therefore, the discordance between the cathodic precharging and CCT in 1Cr-0.2 V steel was attributed to the non-diffusible hydrogen-trapping characteristic of V carbides.

This characteristic of V carbides also makes it difficult to use the [*H*_*E*_] parameter to evaluate the HE resistance, inasmuch as most trapped hydrogens may not contribute to the HE phenomenon. In other words, it would be difficult to determine the resistivity parameters in steels containing V carbides.

### Mo carbides

The above discussions imply the superiority of Mo carbides to Cr carbides from the viewpoint of HE resistance. 1Cr-0.4Mo steel exhibited a significant resistivity parameter because of the low [*H*_*E*_] value and high [*H*_*C*_] value. Regarding the low [*H*_*E*_] value, it contained the lowest amount of hydrogen during the entire cycles of CCT, as shown in Figs 6a and 8. This result may appear inconsistent with the conclusions drawn by Akiyama^5^, who reported a higher amount of hydrogen content in NIMS17 steel (0.94Mo in mass percentage) compared to commercial steels (≤0.16 Mo). This difference arose from the disparity in the Mo compositions between the two studies. Mo carbides observed in NIMS17 steels with 240% higher Mo composition were pronouncedly coarser (~100 nm) and locally distributed as compared to those in 1Cr-0.4Mo steel^14^. This gave rise to a stronger hydrogen absorption in NIMS17 steel^15^.

The high [*H*_*C*_] value, as confirmed in Fig. 4a, stemmed from the nano-sized Mo carbides. The coherent interface of these carbides provided the stable hydrogen trapping in contrast to those of Cr-rich M_7_C_3_ carbides^9^. In addition, the dispersed fine carbides effectively decreased the mean free path of diffusible hydrogen inside a steel. This assisted in the uniform distribution of internal hydrogen, and thus suppressed the localized hydrogen concentration. Such a mechanism successfully explained the variation in HE resistance of Fe-17Mn-(0.5–0.9)C steels with different densities of hydrogen-enriched sites^29^.

Mo and V carbides induced the comparable improvement in HE resistance (i.e. the inhibited NFS reduction) in the present study. However, the mechanisms under the improvement obviously differed depending on the type of carbides. The HE resistance of 1Cr-0.2 V steel arose from the stable hydrogen trapping by V carbides, not from the inhibited hydrogen absorption, as discussed above with Fig. 6. A huge amount of internal hydrogen absorbed in 1Cr-0.2 V steel would cause a significant HE phenomenon at elevated temperatures, which is not an exceptional condition for high-strength steels. In contrast, 1Cr-0.4Mo steel is free from this issue due to the above-mentioned low [*H*_*E*_] value. This is clearly supported by the considerable difference in hydrogen desorption rates at high temperature between 1Cr-0.4Mo steel and 1Cr-0.2 V steel, as shown in Fig. 8. It is also worth noting that Mo addition improves the resistance to corrosive environment other than HE^30,31^. In conclusion, Mo carbide was the ideal alloying element for enhancing HE resistance for applications in corrosive environments.

## Conclusions

A comparison of the effects of Cr, V, and Mo carbides on the HE resistance of tempered martensitic steel under nearly identical conditions has been presented in this study. The alloy-carbide-free 1Cr steel exhibited the lowest activation energy for hydrogen detrapping and the lowest hydrogen-trapping capability preserved at 400–600 °C. This steel showed a severe NFS reduction, suggestive of its low HE resistance. 2Cr steel contained Cr-rich M_7_C_3_ carbides; the incoherent carbide interface and rapid carbide growth gave rise to bad HE resistance as compared to the other steels. Such mechanical deterioration was also supported by the intergranular fracture shown only in this steel. 1Cr-0.2 V steel showed needle-like V carbides with the Baker-Nutting orientation with respect to a ferritic matrix. This steel showed the highest hydrogen-trapping capability and good HE resistance. However, a huge amount of absorbed hydrogen may cause a significant HE phenomenon at elevated temperatures. The non-diffusible hydrogen-trapping characteristic of V carbides led to difficulty in using the resistivity parameter. Finally, 1Cr-0.4Mo steel containing nano-sized Mo carbides was concluded to be the best in terms of improved HE resistance because of the high resistivity parameter, suppressed hydrogen penetration, and low NFS reduction both in SSRT and CCT, as well as the resistance to the most corrosive environments besides the HE phenomenon.

## Methods

In the present study, four specimens based on NIMS17 steel^6^ with a tailored amount of Cr, V, and Mo were prepared to elucidate the effect of various carbides on HE behavior. Table 1 summarizes the chemical composition and denomination of these steels. In a previous study, it was found that undissolved carbides deteriorated the HE resistance^15^. Accordingly, the chemical composition of 2Cr and 1Cr-0.4Mo steels was determined by solubility simulation using the ThermoCalc software with TCS Steel and Fe Alloys Database Ver. 7^32^ to exclude the effect of undissolved Cr and Mo carbides during the austenitizing step.

**Table 1:** Chemical composition and carbide type of the investigated steels.

Sample	Nominal Composition	Actual Composition (mass percentage)								Carbide Type
		C	Si	Mn	P	S	Cr	Mo	V	
1Cr	Fe-0.6C-2Si-0.2Mn-1Cr	0.60	2.01	0.20	<0.003	<0.003	1.01			Cementite
2Cr	Fe-0.6C-2Si-0.2Mn-2Cr	0.60	2.10	0.16	<0.003	<0.003	2.05			Cr carbide
1Cr-0.2 V	Fe-0.6C-2Si-0.2Mn-1Cr-0.2 V	0.60	2.05	0.20	<0.003	<0.003	0.99		0.20	V carbide
1Cr-0.4Mo	Fe-0.6C-2Si-0.2Mn-1Cr-0.4Mo	0.61	2.02	0.20	<0.003	<0.003	1.04	0.39		Mo carbide

Ingots (175 × 165 × 330 mm^3^) were melted in a vacuum arc furnace and then hot rolled to a thickness of 13 mm at 1273–1473 K. 1Cr-0.2 V steel was austenitized at 980 °C for 30 min while the others were austenitized at 920 °C for the same duration. Such conditions were designed considering the significantly lower solubility of V element compared to that of Cr and Mo. The austenitized samples were subsequently quenched to 60 °C in an oil solution to minimize the residual stress, immediately followed by the tempering step at 500–570 °C. The tempering temperatures were tailored to confer similar UTS of ~1.6 GPa on the investigated steels. This assisted in clarifying the effect of each carbide more precisely by minimizing the influence of material strength that significantly affects HE resistance^33^. The tempering time was set as 1 h for all cases. Table S1 summarizes the specific heat-treatment conditions and resultant tensile properties of the investigated materials.

The entire surfaces of the samples were water-abraded using #400- to #1200-grit SiC papers to ensure uniform hydrogen charging. Hydrogen was charged into specimens in an aqueous solution of 0.4% NaOH at room temperature. The charging current of 0.1–30 A∙m^−2^ was applied for 48 h based on the ISO 16573 standard. CCT was performed using the three-stage method^34^; one cycle consisted of the drying stage (50% humidity) for 345 min, wetting stage (98% humidity) for 345 min, and spraying stage (0.5% NaCl solution) for 30 min. The amount of hydrogen inside each sample was measured using the TDS analysis conducted by the gas chromatography and quadrupole mass spectrometry.

Microstructures were characterized using the TEM and 3DAP analysis. The samples for both experiments were prepared using the replica and focused ion beam (FIB) methods. The fracture surfaces were observed by scanning electron microscopy after cleaning the surfaces using acetone.

Tensile properties were evaluated using a hydraulic dynamic testing machine at a strain rate of 5 × 10^−3^ s^−1^ at room temperature. The specimens were machined with a gauge diameter of 6 mm and gauge length of 25 mm. The tensile tests were repeated thrice per condition for data reliability. The NFS was measured by the SSRT at an initial crosshead speed of 5 × 10^−3^ mm∙min^−1^. The SSRT used hydrogen-charged notch specimens with an intensity factor of 4.37 to simulate the screw thread of a bolt (Fig. S4). Prior to commencing the test, these specimens were coated with Zn at a current density of 50 A∙m^−2^ for 5 min to prevent hydrogen from diffusing out of a specimen during the SSRT. The testing time varied from 1 h to 5 h depending on the mechanical strength of material.

## Supplementary information

SI

## Data Availability

The datasets generated during and/or analyzed during the current study are available from the corresponding authors on reasonable request.
