# Supplementary material for: Comparative study on the effects of Cr, V, and Mo carbides for hydrogen-embrittlement resistance of tempered martensitic steel
Source: Sci Rep. 2019 Mar 26;9:5219. doi: 10.1038/s41598-019-41436-2 (PMC6435655; doi:10.1038/s41598-019-41436-2)
Supplement: Supplementary file 1 — SI [file 41598_2019_41436_MOESM1_ESM.docx]

**Comparative study on the effects of Cr, V, and Mo carbides for hydrogen-embrittlement resistance of tempered martensitic steel**

Junmo Lee^1^, Taekyung Lee^2,*^, Dong-Jun Mun^1^, Chul Min Bae^1^, Chong Soo Lee^3,*^

^1^ *Pohang Research Lab, POSCO, Pohang 37877, Korea*

^2^ *School of Mechanical Engineering, Pusan National University, Busan 46241, Korea*

^3^ *Graduate Institute of Ferrous Technology, Pohang University of Science and Technology (POSTECH), Pohang 37673, Korea*

* Corresponding authors:

Prof. T. Lee, Tel: +82-51-510-2985, E-mail: [taeklee@pnu.ac.kr](mailto:taeklee@pnu.ac.kr)

Prof. C.S. Lee, Tel: +82-54-279-9009, E-mail: [cslee@postech.ac.kr](mailto:cslee@postech.ac.kr)

**Table S1**

Heat treatment conditions and mechanical properties of the investigated specimens. The numbers in parenthesis indicate a standard deviation.

| Sample | Austenitizing Temperature (°C) | Tempering Temperature (°C) | | UTS (MPa) | Elongation to Failure (%) |
| --- | --- | --- | --- | --- | --- |
| 1Cr | 920 | 500 | 1589 (9) | | 12.1 (0.2) |
| 2Cr | 920 | 550 | 1612 (10) | | 12.2 (0.1) |
| 1Cr-0.2V | 980 | 570 | 1668 (13) | | 12.4 (0.2) |
| 1Cr-0.4Mo | 920 | 570 | 1638 (4) | | 12.4 (0.3) |


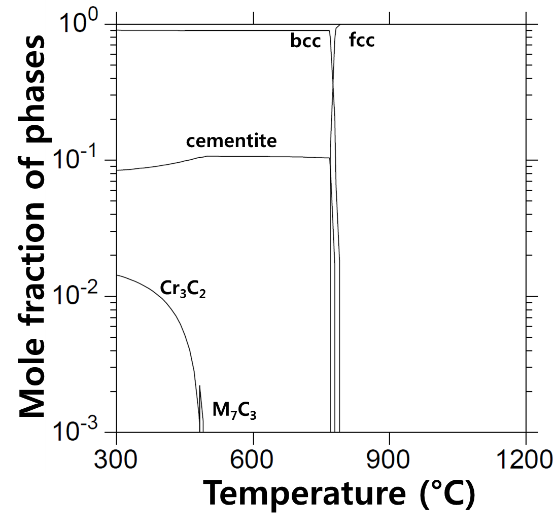

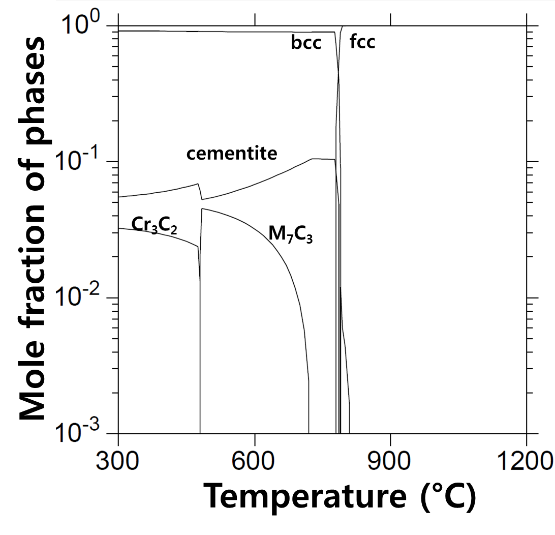


(a) (b)


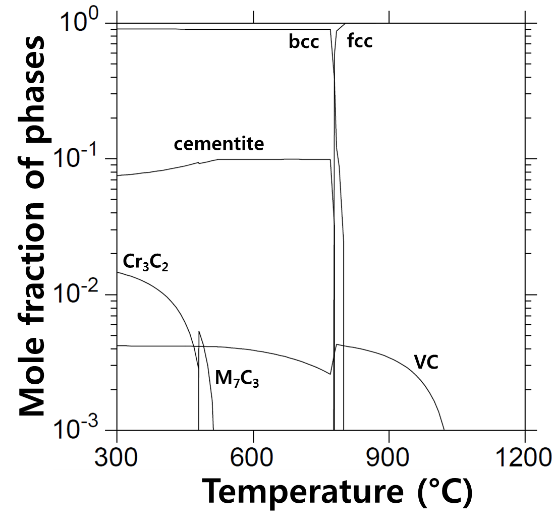

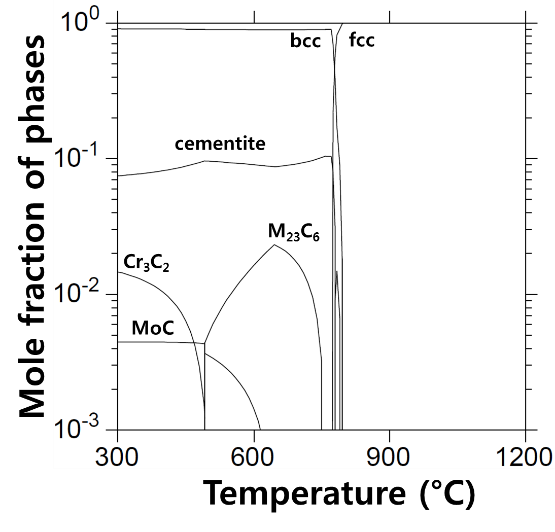


(c) (d)

**Figure S1.** Phase fraction with varying tempering temperature: (a) 1Cr steel, (b) 2Cr steel, (c) 1Cr-0.2V steel, and (d) 1Cr-0.4Mo steel.


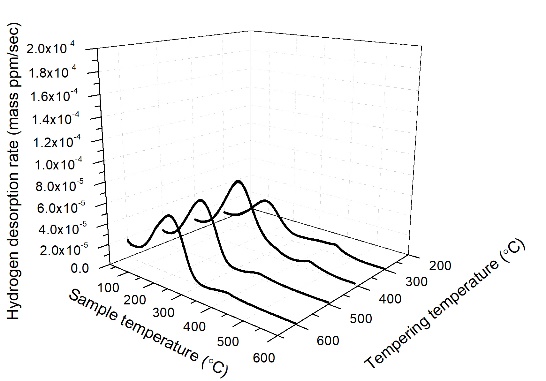

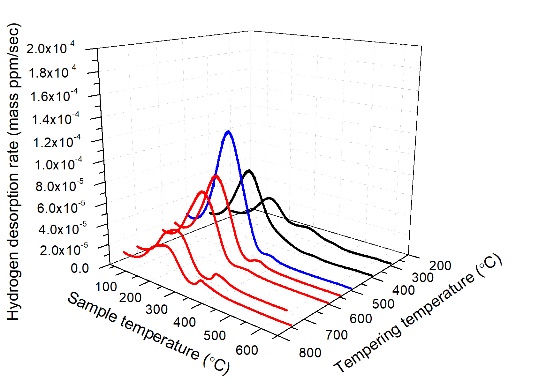


**1Cr Steel**

**2Cr Steel**


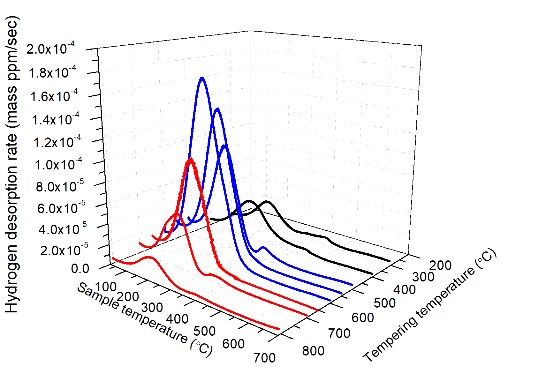

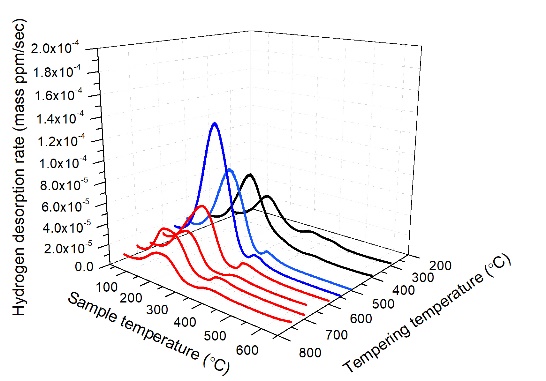


**1Cr-0.4Mo Steel**

**1Cr-0.2V Steel**

**Figure S2.** The change in TDS curves for the investigated steels at various tempering temperatures. Blue and red curves indicate the temperature at which hydrogen trapping increased as a result of carbide precipitation or it decreased because of carbide coarsening, respectively.





**Figure S3.** Arrhenius plots of the investigated steels for calculating the activation energy for hydrogen detrapping.


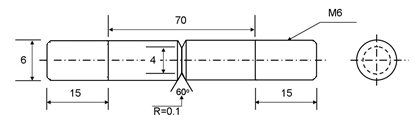


**Figure S4.** Sample dimension for the SSRT performed in this work (number in mm).
